# Role of disorder when upscaling magnetocaloric Ni-Co-Mn-Al Heusler alloys from thin films to ribbons

**DOI:** 10.1038/s41598-018-27428-8

**Published:** 2018-06-14

**Authors:** B. Weise, B. Dutta, N. Teichert, A. Hütten, T. Hickel, A. Waske

**Affiliations:** 1 Leibniz Institute for Solid State and Material Research Dresden, 01069 Dresden, Germany; 2 Institute for Material Science, TU Dresden, 01062 Dresden, Germany; 3 Max-Planck-Institut für Eisenforschung GmbH, 40237 Düsseldorf, Germany; 4 Center for Spinelectronic Materials and Devices, Department of Physics, Bielefeld University, 33615 Bielefeld, Germany

## Abstract

Research in functional magnetic materials often employs thin films as model systems for finding new chemical compositions with promising properties. However, the scale-up of thin films towards bulk-like structures is challenging, since the material synthesis conditions are entirely different for thin films and e.g. rapid quenching methods. As one of the consequences, the type and degree of order in thin films and melt-spun ribbons are usually different, leading to different magnetic properties. In this work, using the example of magnetocaloric Ni-Co-Mn-Al melt-spun ribbons and thin films, we show that the excellent functional properties of the films can be reproduced also in ribbons, if an appropriate heat treatment is applied, that installs the right degree of order in the ribbons. We show that some chemical disorder is needed to get a pronounced and sharp martensitic transition. Increasing the order with annealing improves the magnetic properties only up to a point where selected types of disorder survive, which in turn compromise the magnetic properties. These findings allow us to understand the impact of the type and degree of disorder on the functional properties, paving the way for a faster transfer of combinatorial thin film research towards bulk-like materials for magnetic Heusler alloys.

## Introduction

Thin films are very good model systems to study the properties of functional magnetic materials^1^ when varying their chemical composition^2^ and degree of disorder^3^. Combinatorial growth of thin films allows one to screen large regions of the phase diagram of an alloy very efficiently^4^. Since for the preparation of thin films full control over the processing parameters (e.g. deposition temperature and - rate) is required, the homogeneity and degree of order in thin films is typically much better than in polycrystalline bulk materials. However, for a number of functional magnetic materials - including magnetocaloric - an upscaling step towards more bulk-like structures is required after a new material composition with promising properties has been identified.

Magnetocaloric Heusler alloys have gained attention recently due to their versatile properties upon slight variation in the chemical composition^5,6^. In this material class, the martensite to austenite transition is associated with large changes in entropy and correspondingly also large adiabatic temperature changes, which form the basis of a magnetic cooling cycle^7^. Within the group of magnetocaloric Heusler alloys, Ni-Co-Mn-Al is a promising alloy system for magnetic refrigeration, as its components are abundant, rare-earth free and non-toxic. In thin films of Ni-Co-Mn-Al, an entropy change of up to ∆S_mag_ = 7.2 Jkg^−1^ K^−1^ for a magnetic field change of ∆µ_0_H = 2 T was observed^8^. As the cooling power achievable in a cooling process scales with the volume of magnetocaloric material used, a certain mass of active material is required. In fact, currently existing magnetocaloric devices are operating with porous magnetocaloric regenerators^9^ of typically some centimetres edge length to exchange heat. Hence for the application of the Ni-Co-Mn-Al Heusler alloy for refrigeration, an upscaling step is needed. Melt-spun ribbons have a favourable geometry with a large surface to volume ratio, comparable to very thin plates. However, the melt-spinning process gives less control over the material processing, with consolidation routes and timescales different than in thin film preparation. In particular, a larger degree of disorder and inhomogeneity is expected in the ribbons compared to thin films.

Interestingly, the presence of chemical disorder in melt-spun ribbons opens up the possibility of tuning this degree of freedom to achieve improved functional properties. Several attempts have been made in the last few years to deliberately modify the degree of disorder and study the corresponding effect on the transition temperatures^10–14^. The most established approach to tune the atomic ordering in the experiments is to anneal the samples at temperatures where the ordered phases are thermodynamically stable. For instance, the effect of annealing temperature on the martensitic transformation and the magnetic properties has been studied intensively in Ni-Mn-Ga^10,11^ and Ni-Co-Mn-Ga^12,13^ alloys. Recent experiments even suggest the possibility of improving the magnetocaloric effect with optimal annealing^15,16^.

In spite of its huge importance, the understanding of the impact of chemical ordering on the magnetocaloric properties, however, is rather limited. A particularly missing link in state-of-the-art investigations is the atomistic picture of the experimentally annealed samples. Using first principles calculations, Schleicher *et al*. have demonstrated recently the impact of partial chemical disorder on the electronic structure and the magnetic properties in Ni-Co-Mn-Ga Heusler alloy^17^. While this study highlights the relevance of chemical disorder, a connection between the annealing time and the evolution of the atomic configurations is not understood thus far. In the present work, we investigate experimentally the impact of chemical disorder present in the melt-spun ribbons of Ni-Co-Mn-Al alloy on the magnetocaloric properties entropy change ΔS_T_ and thermal hysteresis (δT_hyst_). Our observations are supported by first principles calculations, which provide a qualitative picture of the underlying disordered atomic configurations and their subsequent impact on the above mentioned magnetocaloric properties.

## Results

### Experimental results

**Figure 1:**
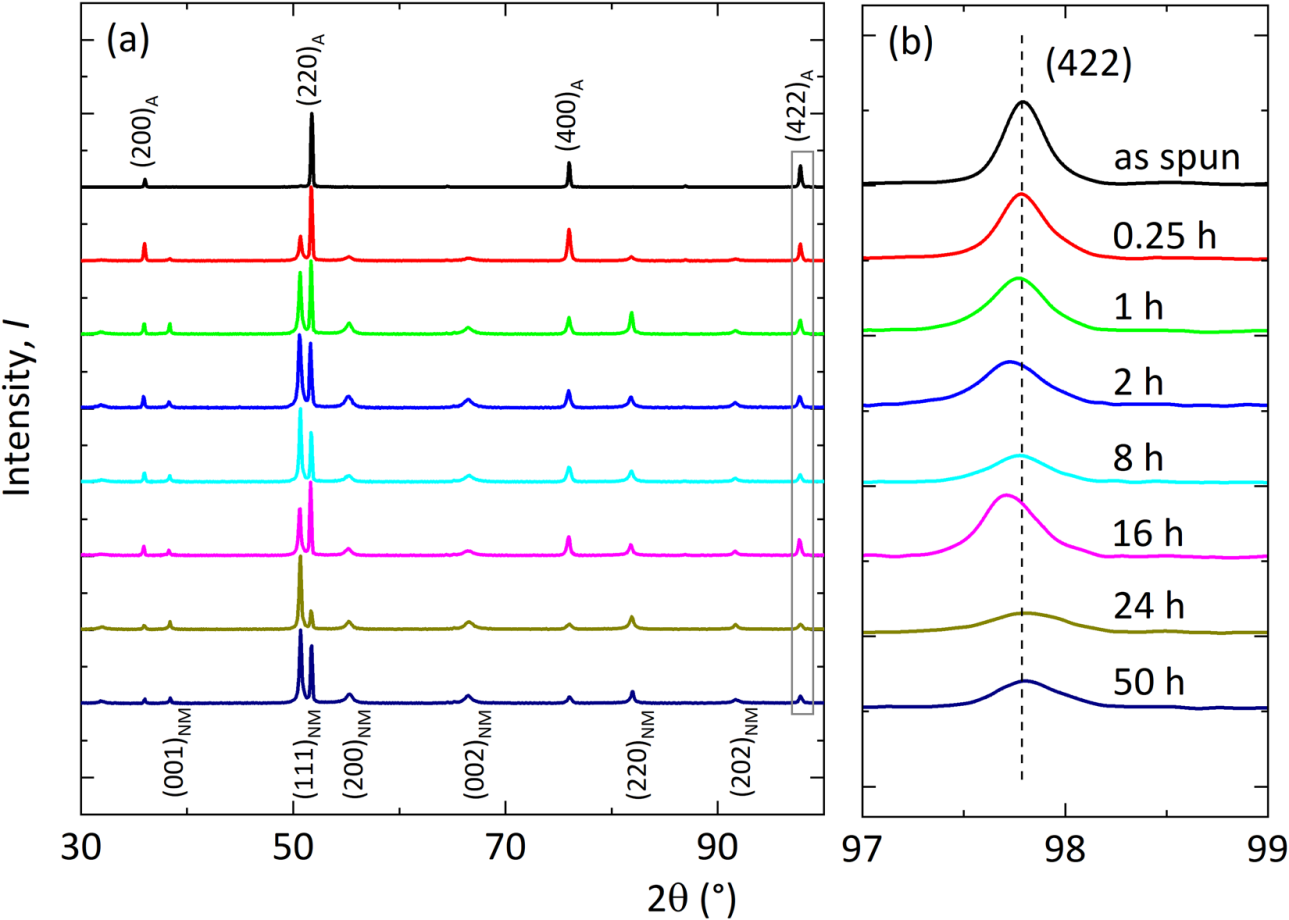
Results of X-ray diffraction at room temperature for different durations of annealing at 850 °C. Right: Zoom view of a (422) austenite peak. Austenite peaks are indicated with A-subscripts, non-modulated martensite peaks with NM.

As it was previously shown in several studies^15,16,18–20^, annealing can improve the magnetocaloric properties of Heusler alloys. Compared to bulk Heusler alloys, reduced annealing times of around some minutes to hours are typical for melt-spun geometries^6^, owing to their large surface to volume ratio. Figure 1 shows the diffractograms of as-spun and heat treated Ni-Co-Mn-Al ribbons. The as-spun ribbon shows a purely austenitic structure, whereas the heat treated samples have martensitic peaks as well. Even after a short heat treatment of t = 0.25 h the formation of martensite coexisting with the austenite phase was observed. The sharpness of the peaks indicate a high level of crystallinity. There are no odd-numbered reflexes visible and therefore a B2-ordering is present^21^ in the annealed samples, as well as in the Ni-Mn-Co-Al thin film (see also Teichert *et al*.^8^). Suppression of L2_1_ order has also been reported earlier for Ni-Mn-In melt-spun ribbons^22^. Furthermore, no difference in the X-ray diffraction pattern between the wheel and free side of the ribbons is observed.

A peculiar feature is the shift to slightly smaller 2θ values, i.e. a larger lattice parameter for the austenite peaks 400 (not shown) and 422 (see Fig. 1(b)) with increased duration of annealing up to 16 h. For annealing times longer than 16 h, this shift in lattice parameter however almost reverts to its initial position.

The crystallographic ordering of as-spun and annealed ribbons as a function of annealing time was calculated using eq. 1. The ordering parameter as a signature of B2 ordering indicates that order drastically increases for all annealed samples (S(B2) = 0.76 +/− 0.04) compared to the as-spun state (S(B2) = 0.55).

**Figure 2:**
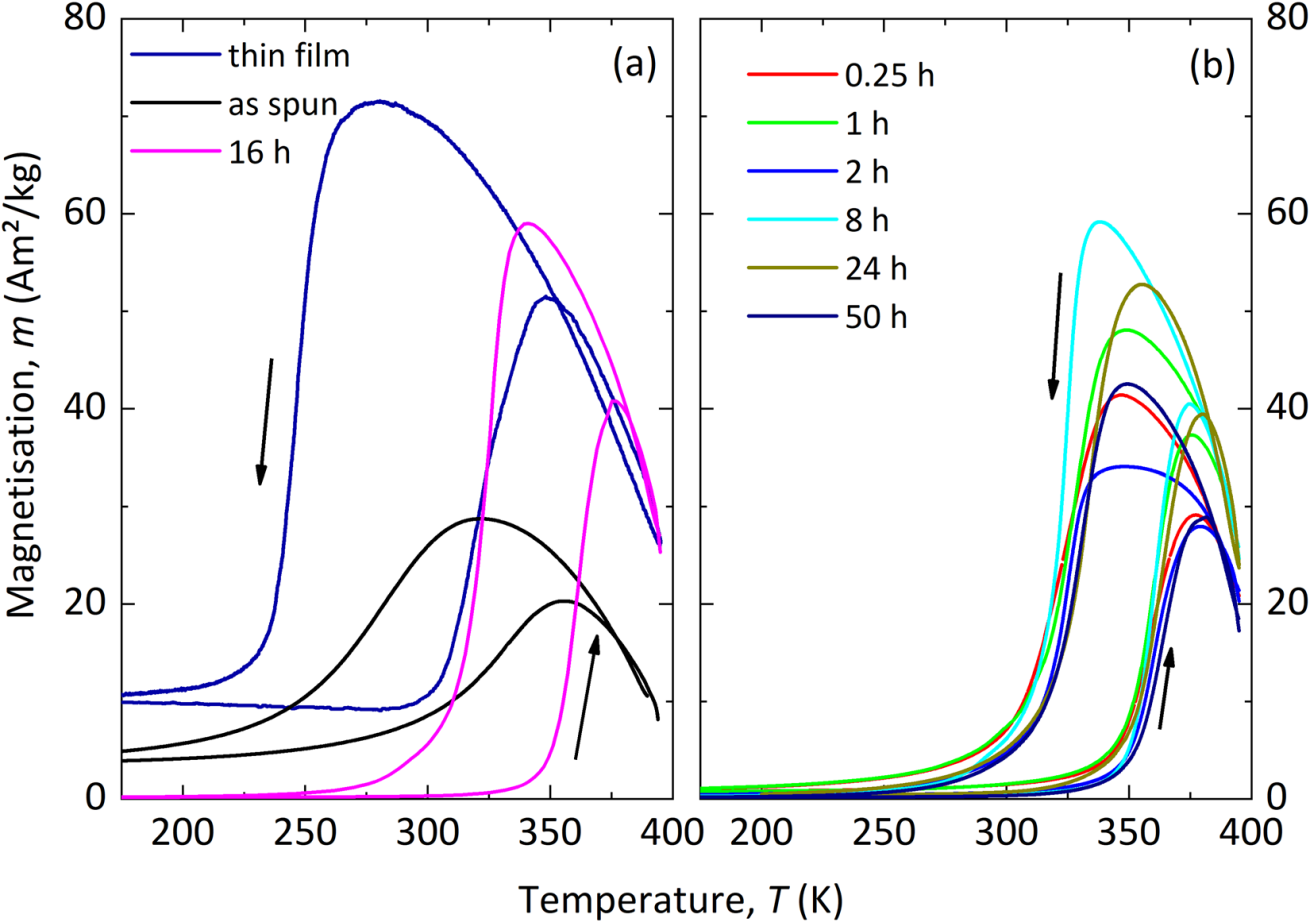
The free standing thin film and the melt-spun ribbon with the influence of annealing on the magnetisation as function of temperature in a magnetic field of µ_0_H = 10 mT. The comparison of the thin film sample, the as-spun ribbon and the optimized heat treatment is seen on the left, whereas the results of the different heat treated ribbons are shown on the right.

In Fig. 2 the temperature dependent magnetisation and in Table 1 the extracted values from the magnetisation measurements are shown. The free standing thin film (Ni_39.4_Co_9.2_Mn_32.3_Al_19.1_) has a larger hysteresis and higher magnetisation compared to both the as-spun and annealed ribbons (Ni_40.9_Co_10.8_Mn_29.3_Al_19.1_). It can be seen that the magnetisation of the austenitic state increases up to an annealing time of 16 h and starts to reduce for longer annealing times. With increasing duration of the heat treatment, both the martensitic (T_M_) and austenitic transition temperature (T_A_) determined from the inflection points of the M(T) curve are shifted to higher temperatures, while the T_C_ remains constant at around T = 405 K. The temperature shift for the longest annealing step compared to the as-spun state is approximately 31 K for T_A_ and 50 K for T_M_ (see Supplementary Fig. S1). Wu *et al*.^23^ showed a similar shift of circa 50 K in Ni-Mn-Sn-Heusler ribbons by annealing for 15 min. The width of the transition is characterized by the difference of start and finish temperatures of martensite and austenite (δA = A_f_ − A_s_ and δM = M_s_ − M_f_)^24^. For the melt-spun ribbons, compared to the as-spun material, it is reduced by 60 K for the martensite and by 35 K for the austenite transition by annealing and therefore it is comparable to the free standing thin film (see Table 1).

**Table 1:** The start, transition and finish temperatures and the transition width for the austenite and martensite transition for the thin film and the different annealing times of the melt-spun ribbons.

		A_s_ (K)	T_A_ (K)	A_f_ (K)	δA (K)	M_s_ (K)	T_M_ (K)	M_f_ (K)	δM (K)
thin film		304.4	320.0	345.3	40.9	270.6	246.0	234.1	36.5
as-spun		292.5	332.0	357.5	65.0	328.6	283.0	236.9	91.7
Annealing time, *t* (h)	0.25	345.2	358.0	374.7	29.5	344.8	323.0	304.2	40.6
	1	347.6	360.0	373.5	25.9	343.2	328.0	310.2	33.0
	2	350.8	359.2	373.6	22.8	330.0	325.3	312.4	17.6
	8	351.5	361.0	371.4	19.9	334.5	325.0	313.8	20.7
	16	351.0	361.0	373.9	22.9	338.2	325.0	311.6	26.6
	24	350.0	363.2	379.1	29.1	354.9	333.3	319.0	35.9
	50	351.0	363.1	379.9	28.9	349.8	333.3	314.6	35.2

Moreover the hysteresis (δT_hyst_ = T_A_ − T_M_) of the transition decreases from δT_hyst_ = 49 K in the as-spun state, to around δT_hyst_ = 34 K +/− 3 K for the annealed samples. The free standing thin film has a hysteresis of δT_hyst_ = 74 K.

**Figure 3:**
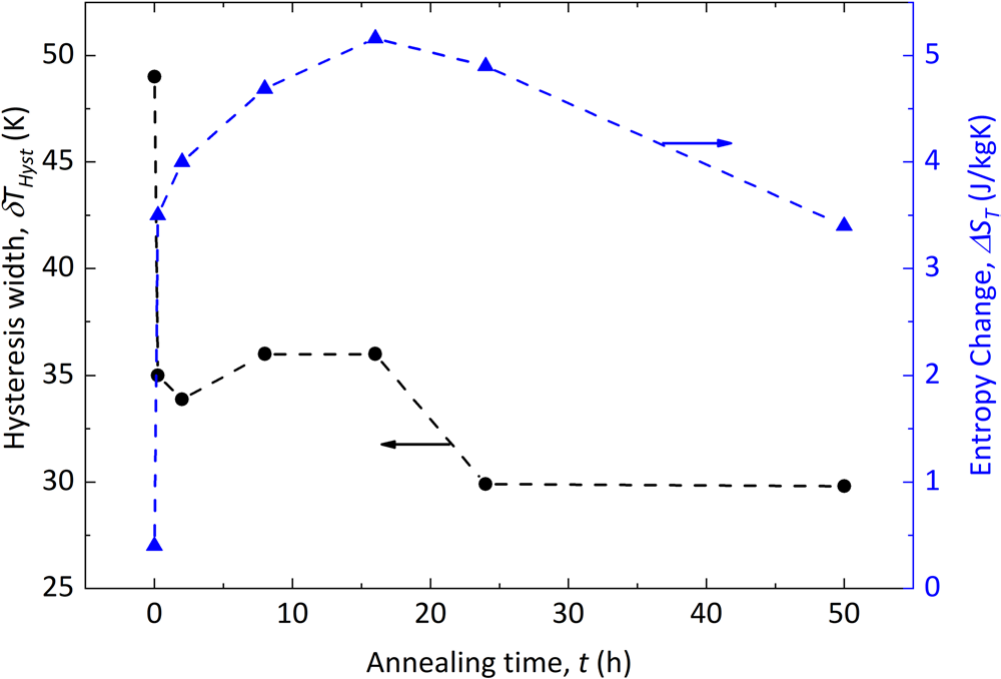
Thermal hysteresis (black dots) and the maximum entropy change for a field change of ∆H = 2 T (blue triangles) of the ribbons as a function of annealing time.

As can be seen in Fig. 3 both the entropy change and the hysteresis width are changed drastically by annealing. A large increase of the entropy change is achieved already after the very first annealing step (t = 0.25 h). Upon further annealing, a further increase of ∆S_T_ is noted until after 16 h of heat treatment at T = 850 °C a maximum entropy change of ∆S_T_ = 5.2 Jkg^−1^ K^−1^ is observed. For longer heat treatments, the entropy change is reduced again. The thin film exhibits an entropy change of ∆S_T_ = 7.2 Jkg^−1^ K^−1^ (Δµ_0_H = 2 T). This optimized entropy change value is higher compared to bulk Ni-Co-Mn-Al material^24^ and similar to Ni-Co-Mn-Sn melt-spun ribbons^25^ with shorter annealing time. The thermal hysteresis shows a two-step behaviour; even for short annealing time a large reduction of δT_hyst_ is observed and for annealing times over 16 h a further reduction of δT_hyst_ is measured. For similar chemical composition in bulk material^24^ and ribbon samples^25^ δT_hyst_ values below 10 K are reported.

From heat capacity measurements and the isothermal entropy change, the adiabatic temperature change was calculated according to eq. 3. In a field change of ∆µ_0_H = 2 T a temperature change of up to ∆T_ad_ = −1.9 K (∆T_ad_ = −0.9 K for 1 T) is expected.

### Numerical simulation results

#### Influence of Co doping

**Figure 4:**
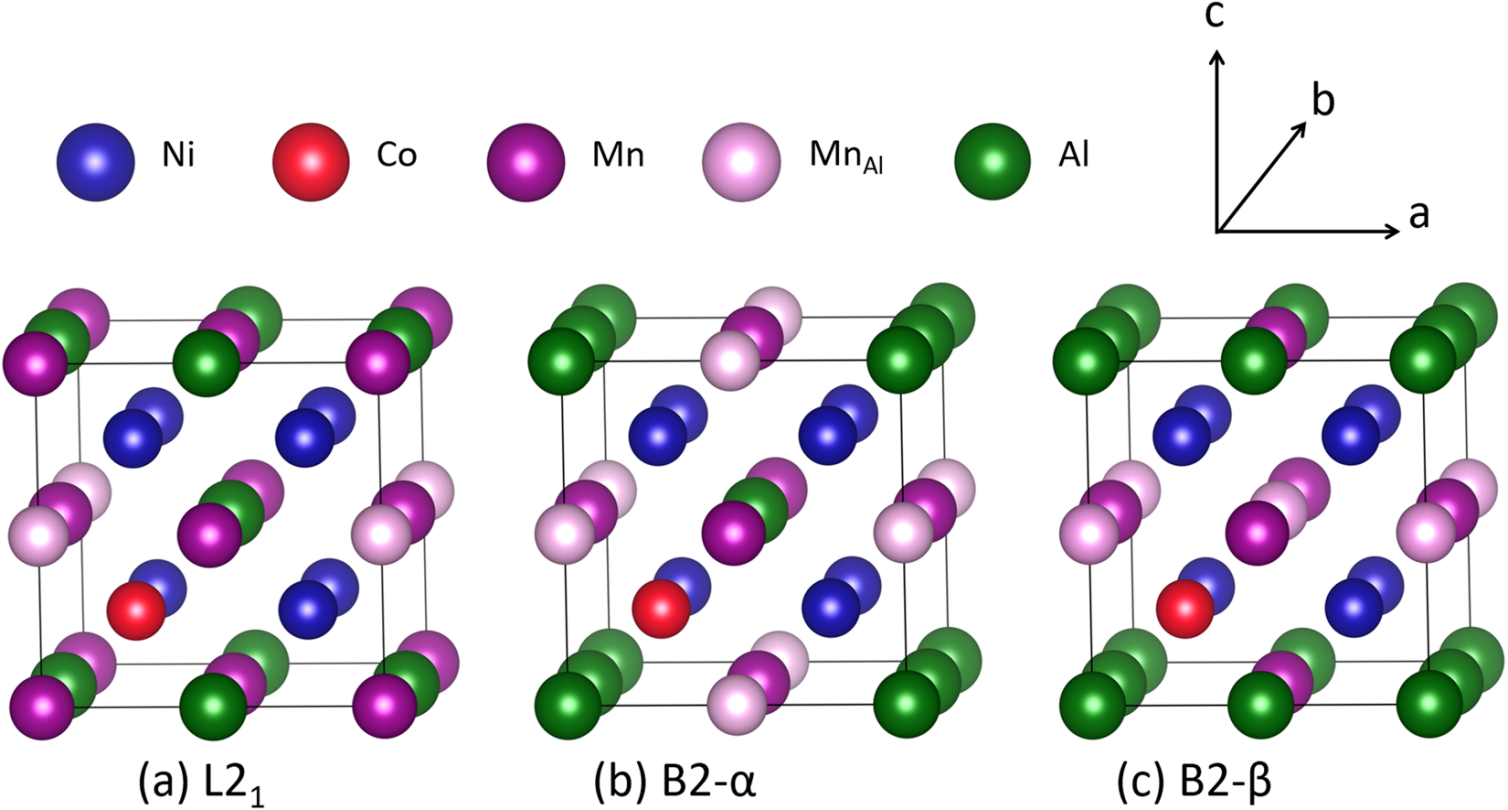
Complete set of non-equivalent atomic configurations of Ni_43.75_Co_6.25_Mn_31.25_Al_18.75_ in a 16 atom unit cell that obey at least B2 order. (**a**) One sublattice is filled by Mn, corresponding to a L2_1_ order; (**b**,**c**) two configurations with a 2:3 distribution of Mn atoms over two sublattices, corresponding to a B2 disorder.

The austenite phase (*c*/*a* = 1.00) of stoichiometric Ni_2_MnAl has a similar crystal structure as Ni_2_MnGa (space group ${\rm{Fm}}\bar{3}{\rm{m}}$) with Ni atoms occupying two out of the four sub-lattices, while Mn and Al atoms fill the remaining two sub-lattices, respectively (L2_1_) or jointly (B2). To handle the complete configurational complexity, we have used a 16-atom unit cell for the theoretical calculations (see Fig. 4). One excess Mn (Mn_Al_) and one Co atom are introduced into the system at the cost of Al and Ni, respectively. In spite of the limitations imposed by the size of the unit cell, the chosen composition for the theoretical calculations, i.e., Ni_43.75_Co_6.25_Mn_31.25_Al_18.75_ closely matches the actual experimental composition.

**Figure 5:**
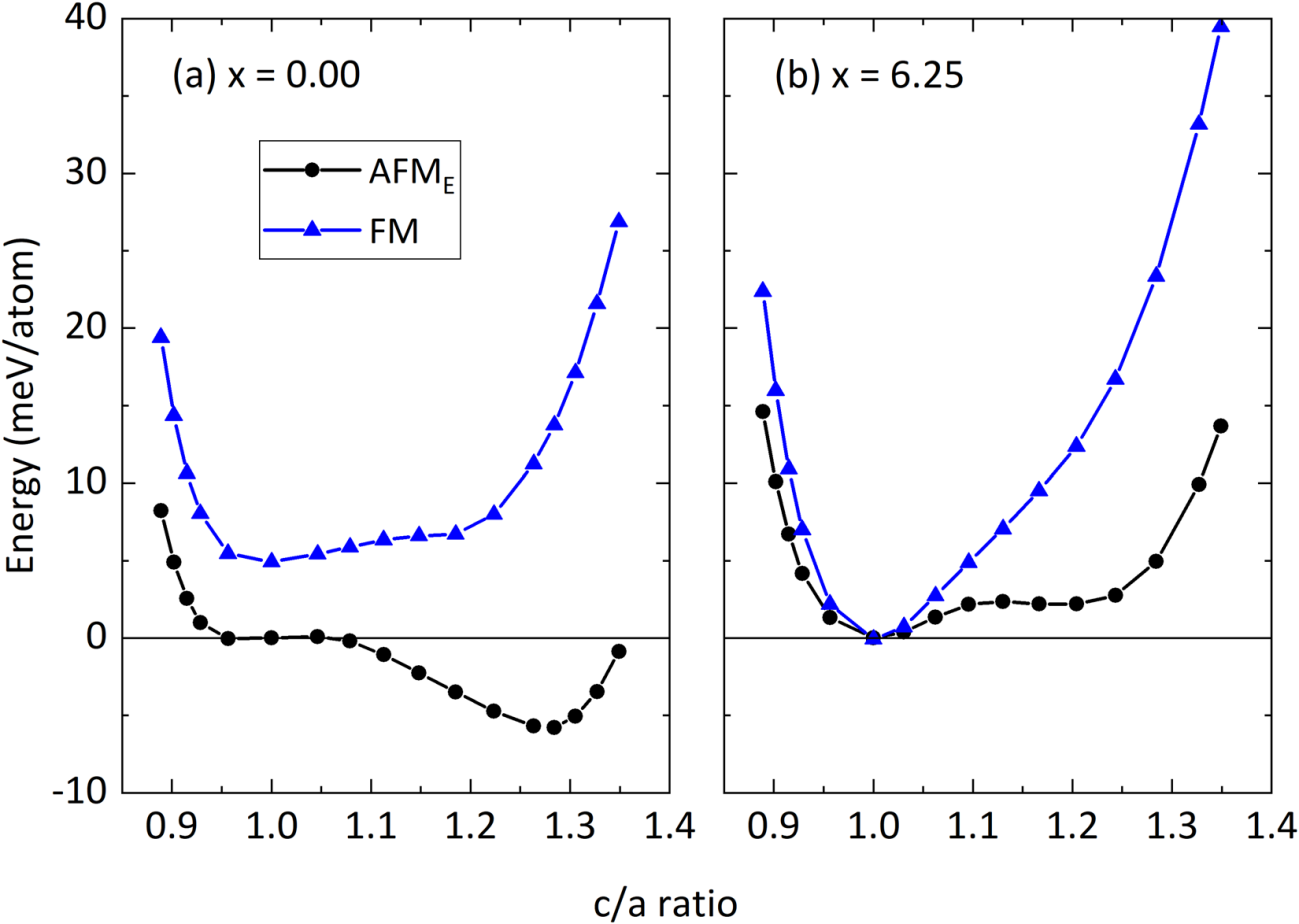
Influence of Co doping on the magnetic ground state of Ni_50−x_Co_x_Mn_31.25_Al_18.75_. The total energy vs. c/a ratio for the ferromagnetic (FM, blue data points) and the antiferromagnetic (AFM_E_, black data points) states for (**a**) x = 0.00 and (**b**) x = 6.25 are shown.

Already in the fully ordered L2_1_ austenite phase of Ni_50−x_Co_x_Mn_50−y_Al_y_, the amount of Co determines whether the spins of the excess Mn_Al_ atoms occupying the Al sub-lattice are aligned in the same direction (FM state) or in the opposite direction (AFM_E_) to those of the host Mn spins. Substitution of Co for Ni leads to strong ferromagnetic exchange interactions between Co and the neighbouring magnetic atoms, which influences the ground state magnetic structure of Ni_50−x_Co_x_Mn_50−y_Al_y_^26^. Figure 5 shows the energy surfaces as a function of *c*/*a* ratio in both the magnetic states (FM and AFM_E_) of Ni_50_Mn_31.25_Al_18.75_ (Fig. 5(a)) and Ni_43.75_Co_6.25_Mn_31.25_Al_18.75_ (Fig. 5(b)). In the case of Ni_50_Mn_31.25_Al_18.75_, the AFM_E_ state is lower in energy for the entire range of *c*/*a* ratios. While a shallow minimum can be seen close to the cubic structure, the ground state is a tetragonal structure with *c*/*a* = 1.28, indicating that the system will undergo a martensitic transition^27^.

Unlike in Ni_50_Mn_31.25_Al_18.75_, we find in Ni_43.75_Co_6.25_Mn_31.25_Al_18.75_ a stable FM austenite phase, which is almost degenerate with the AFM_E_ state. This indicates that the amount of Co substituted for Ni is sufficient to change the nature of the magnetic state in the cubic austenite phase of the present system, but not in the tetragonally deformed phase. An interesting observation in Fig. 5(b) is, however, that even the AFM_E_ state is only metastable upon this deformation. This indicates that the Co substitution stabilizes the cubic L2_1_ austenite phase down to 0 K and hence Ni_43.75_Co_6.25_Mn_31.25_Al_18.75_ does not undergo a martensitic transition. This observation is consistent with our previously calculated results^26^ for Ni_43.75_Co_6.25_Mn_37.5_Al_12.5_, which showed a martensitic transition at a lower temperature than that of Ni_50_Mn_37.5_Al_12.5_.

While we have established that Co reduces T_M_, our theoretical results for Ni_43.75_Co_6.25_Mn_31.25_Al_18.75_ in Fig. 5(b) do not describe the experimental situation, which clearly revealed a martensitic transformation (cf. Fig. 2) in Ni_40.9_Co_10.8_Mn_29.3_Al_19.1_, i.e. for an even higher Co-content. This indicates that chemical disorder, which is known to influence the martensitic transition^3^, plays an important role in the present alloy system. Indeed, our order analysis using S(B2) indicates B2 ordering in the present alloy, which is in agreement with other studies^8,28,29^. Therefore, the impact of mixing the Mn and the Al sub-lattices on the energy surface is discussed next.

#### Impact of disorder

**Figure 6:**
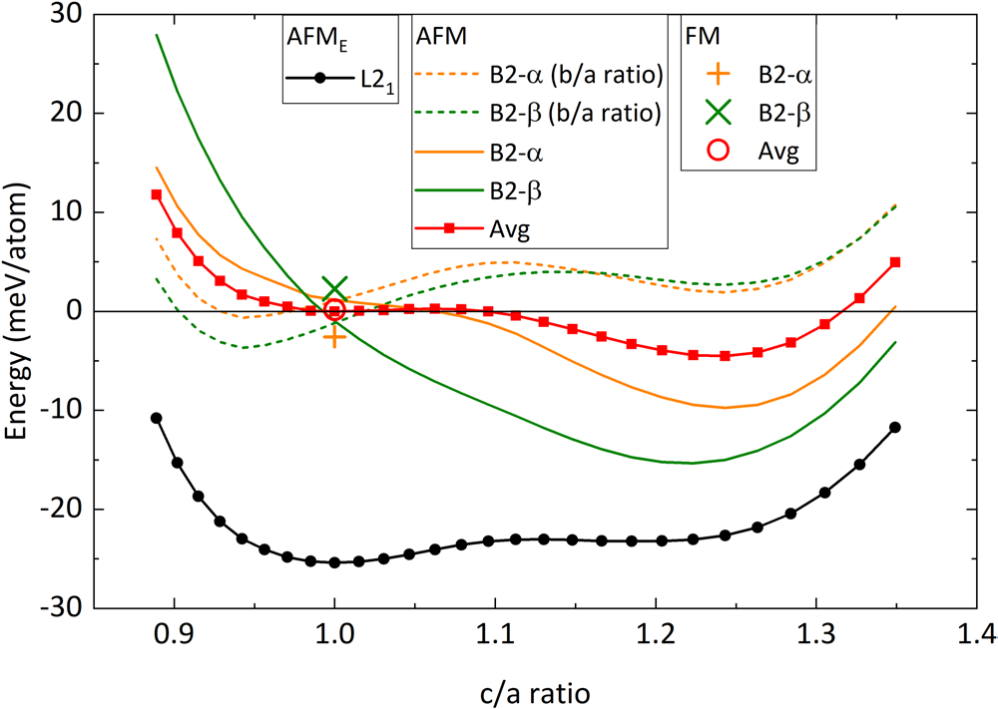
Change in energy as a function of tetragonallity for the different atomic configurations studied for Ni_43.75_Co_6.25_Mn_31.25_Al_18.75_. The structures and crystallographic directions are given in Fig. 4.

The B2 crystal structure has two distinct sub-lattices. For the case of Ni_43.75_Co_6.25_Mn_31.25_Al_18.75_ alloy, Ni and Co atoms occupy one sub-lattice, while Mn and Al atoms share the other sub-lattice. In the 16-atom unit cell chosen for the present study, there can only be two non-equivalent B2 atomic configurations (with and without full Mn layers), named configuration B2-α and B2-β, which are illustrated in Fig. 4(b and c). The energetics of the tetragonal distortion shows a remarkable dependence on the atomic configuration and the direction of the distortion (*c*/*a* vs. *b*/*a*) with qualitatively different trends (Fig. 6). An average over all possible configurations and directions (within the 16 atom cell), however, yields a pronounced minimum for the martensitic phase with *c/a* ≈ 1.25. This clearly indicates that the intermixing of the Al and the Mn atoms in one sub-lattice in the B2 structure has a strong impact on the martensitic transformation. In other words, the martensitic transition observed at the experimental composition Ni_40.9_Co_10.8_Mn_29.3_Al_19.1_ happened only because of this chemical disorder.

The impact of Co on the magnetic state of the B2 structure is qualitatively similar to that of the L2_1_ structure. Without Co, the nearest neighbour Mn_Al_-Mn interaction is antiferromagnetic in nature for all configurations and distortions. However, the presence of Co can change the magnetic state as shown in Fig. 6. In the cubic austenite phase, the FM state is lower in energy for B2-α, whereas the AFM_E_ state is lower for B2-β. The change of the magnetic state in the austenite phase due to Co substitution also has important consequences for the volume of the unit cell and hence the lattice parameter, which is experimentally accessible. The calculated equilibrium volumes for the austenite and martensite phase for both of the configurations are shown in Table 2.

**Table 2:** Equilibrium lattice constant, volume and magnetic state in the cubic austenite phase and in the tetragonal martensite phase (c/a ≈ 1.25) for the two different atomic configurations B2-α and B2-β studied.

Configuration	Austenite			Martensite	
	Magnetic state	Lattice constant (Å)	Volume (Å^3^/atom)	Magnetic state	Volume (Å^3^/atom)
B2-α	FM	5.805	12.226	AFM_E_	12.044
B2-β	AFM_E_	5.786	12.106	AFM_E_	12.012

There are two key results from Table 2:

1. In the austenite phase, the FM state requires a larger volume than the AFM_E_ state.
2. The FM austenite to AFM_E_ martensite transition (e.g., B2-α) is associated with a larger volume reduction than the AFM_E_ austenite to AFM_E_ martensite transition (e.g., B2-β).

Based on these two results, we can qualitatively explain the experimentally observed changes of the lattice parameters for the austenite phase after different annealing times (see Fig. 1).

## Discussion

Our experimental and numerical findings suggest that the annealing of magnetocaloric Ni-Co-Mn-Al proceeds in the following three steps with increasing annealing time:

1. Upon very short annealing (t = 0.25 h), an initial disorder-order transformation from the A2 phase to B2 phase takes place, which is characterized by a jump in the order parameter S(B2) from 0.55 to 0.76 and a corresponding step-like increase of the magnetisation and entropy change while simultaneously the thermal hysteresis decreases sharply.
2. For annealing times between t = 1–16 h, the B2 austenite phase (*c*/*a* = 1.0 in Fig. 6) is a mixture of different disordered configurations. With increasing the annealing time from 1 h to 16 h, configurations like B2-β seem to transform to configurations like B2-α. Such an increase in the proportion of B2-α, which are in the FM state in the austenite phase, can explain the increase of volume and hence lattice parameter seen in our X-ray diffraction experiments (cf. Fig. 1) for the 2 h to 16 h annealed samples. As a consequence, the martensitic transition between a FM austenite phase and an AFM martensite phase will imply a larger change in volume, which will increase hysteresis again. This is most likely why the hysteresis does not decrease faster for annealing times t = 8 h and 16 h (see Fig. 3). Based on our argument that more B2-α-like configurations will be stabilized in the above-mentioned annealing time, the magnetisation of the austenite phase and the magnetic entropy change (roughly based on the difference between austenite and martensite magnetisation) should also increase in this period. In the experiments, exactly these trends are evident. These findings are consistent with an increase in magnetisation with increasing B2-order found in Ni-Mn-In systems^30^.
3. For longer annealing, another ordering process is apparently active. The evaluation in Fig. 6 indicates an example of a possible scenario, namely the B2-β configuration with its energy minimum for b/a ≈ 0.95. The additional (local) distortion in such a scenario could explain higher energy barriers and, therefore, longer annealing times. The decreased relevance of B2-α by this or other mechanisms could explain the decrease of net magnetisation of the austenite for 24 h- and 50 h-annealed samples, the decrease of the entropy change, the reduction in volume of the austenite phase (cf. Fig. 1), and therefore the lowering of the hysteresis effect. A decrease in the hysteresis width has in fact been observed in experiments in case of 24 h- and 50 h-annealed sample, while a direct signature of tetragonal distortion for these two samples is lacking in the XRD data (cf. Fig. 1). It is likely that the distortions are local phenomena not detectable by XRD, but sufficient as nucleation centres for the martensitic transition.

While the presented numerical simulation and the postulated relevance of B2-α-like configurations are in correspondence with all experimental results, the T = 0 K energetics provided in Fig. 6 with energy differences of a few meV for the austenite is insufficient to rigorously explain the driving force for the B2-α stabilization at the annealing temperature of 850 °C. Configurational entropy will certainly play the key role since the B2-β structure with its fully occupied Mn layers has a higher degree of order than the B2-α structure. However, a substantially larger amount of configurations in supercells containing many more atoms would be required, to achieve a reasonable statistics for such an analysis. Together with the fact that also magnetic disorder and lattice vibrations would need to be taken into account, that goes beyond the scope of this study.

The dependence of the magnetocaloric properties of NiMn-based Heusler alloys on the annealing procedure has been analysed by numerous works^15,16,18,20^. Particularly the response to very short annealing time (<1 h) was studied for a number of magnetocaloric Heusler alloys^16,18,19^, and explained with the transition from A2 to B2 ordering, like in step 1 of our 3-step scenario above. Zhang *et al*.^16^ observed that an optimal annealing time can be found, for which the entropy change is maximized, while at the same time the hysteresis is decreased. Here, the decrease of the entropy change (and other related quantities) for annealing times longer than the optimal one was explained with an increasing deviation of the chemical composition in the “over-annealed” samples. In Zhang’s work, this is in correspondence with a very slight broadening of the transition width^16^, which we also observe in our samples (cf. Table 2). Whether decomposition of the material like suggested by Entel *et al*.^3^ plays a role for our composition is unclear. In the diffractogram (cf. Fig. 1), no additional phases appear for long annealing up to 50 h. For lower annealing temperature (377 < T < 477 °C) under applied field for Mn-rich Ni_2_MnX (X = In, Sb, Sn or Ga), decomposition into ferromagnetic Ni_2_MnX and antiferromagnetic NiMn, leading to so called shell ferromagnetism, is observed^31,32^. We do not expect decomposition in our samples due to the lower annealing time and the Co alloying.

In comparison to our findings for a thin film of Ni-Co-Mn-Al^8^, which stimulated the current work, the optimally annealed ribbons show a similar magnetisation and correspondingly, also a similar entropy change as their thin film counterparts. Also the transition width is comparable in ribbons and thin films, indicating that the chemical order achieved in optimally annealed ribbons is very similar to the thin film material. This proves that for optimal annealing conditions, ribbons can reproduce the behaviour that is otherwise only achieved in thin films with very precise control of the (slow) growth. The thermal hysteresis is even substantially decreased in the annealed ribbons (30 K) compared to the freestanding thin films (71 K). Similar to findings by Bruno *et al*.^25^, which show that the thermal hysteresis δT_hyst_ is reduced by annealing (t = 2 h) of melt-spun ribbons due to the decreasing number of grain boundaries, we see a reduction of δT_hyst_ by short time annealing and a further reduction for longer annealing (cf. Fig. 3).

Our theoretical findings suggest a tetragonal distortion of the cubic lattice already in the austenite state when prolonged annealing is applied. These distortions, though only be present locally, might well act as a precursor for the martensite transformation, easing the transition by providing nuclei and hence reducing the hysteresis as shown by Niemann *et al*.^33^. Indications of local atomic relaxations were previously observed in experiments by Bhobe *et al*.^34^ and Lobo *et al*.^35^ for Mn-rich Ni-Mn-Sn and Ni-Mn-In alloys, respectively. These local relaxations were caused by the atomic size difference between excess Mn atoms occupying the Sn or In sub-lattice and the host Sn or In atoms. While similar relaxation effects are also present in our Ni-Co-Mn-Al alloy for both L2_1_ and B2 structures, the additional chemical disorder in the B2 structure is the main cause for the tetragonal distortion as obtained in our theoretical calculations. The small effect of atomic relaxations on the qualitative nature of the energy surface for the B2 structure has been confirmed by comparing calculations without and with relaxation effects. Furthermore, it is known from Ni-Mn-Co-Sn systems that atomic disorder in melt-spun ribbons decreases the thermal hysteresis by improving the lattice coherence between martensite and austenite phases^22^. This facilitation of the martensite nucleation is also reflected by a higher martensite start temperature M_S,_ i.e. an earlier onset of martensitic transformation for longer annealed samples, as shown in our experimental results for t = 24 h and 50 h in Table 1. A reason for the difference in the transition temperatures and –width, are the different nucleation mechanisms of thin films compared to melt-spun ribbons. In thin films the surface is flat and the material is similar to a single crystal^33^, whereas melt-spun ribbons have an uneven shaped surface. This surface roughness can act as a preferential nucleation site^36^ and therefore reduce the hysteresis. In addition, the thin films are only prepared at 500 °C compared to an annealing temperature of the ribbons of 850 °C, this is probably not enough to induce the local distortion in the films. Furthermore it is known, that in Heusler systems the martensite transition is initiated by nucleation, whereas the austenite transition is limited by growth only^37^.

The final goal of finding compositions with promising magnetocaloric performance in thin films and then transferring these candidates to ribbons is a significant step towards designing a material that can provide a large adiabatic temperature change. For the optimally annealed ribbons, we determined a value of ∆T_ad_ = −1.9 K for an indirect measurement. This is comparable to the values for bulk material (∆T_ad_ = −1.0 K) with a similar composition^24^, when taking into account that those direct cyclic measurements are typically providing a more realistic value than the (over-) estimations obtained from indirect measurements (see also Gottschall *et al*.^7^).

## Conclusion

We have shown that it is possible to reproduce the excellent properties of highly ordered magnetocaloric Ni-Co-Mn-Al thin films with melt-spun ribbons if an appropriate heat treatment is applied. The optimal annealing time has to be long enough to induce the disorder-order transformation from A2 to B2, but short enough to avoid the formation of “selective” remaining disorder leading to decreased performance. This principle can most likely be applied to a wide range of magnetocaloric Heusler alloys. For our composition (Ni_40.9_Co_10.8_Mn_29.3_Al_19.1_), the optimal annealing time is between 16 and 24 h, which leads to values of the magnetisation and entropy change comparable to the thin film, while significantly reducing the thermal hysteresis in the ribbons compared to thin films. We argue that the reduction of hysteresis is owed to the tetragonal distorted austenite phase appearing upon prolonged annealing, which acts as a nucleation centre for the martensitic transition. Furthermore, the rough surface of the ribbons, compared to a perfectly smooth surface of the thin film, provides some opportunities for compensating strain and even for preferential nucleation at convex surface^36^, which both decrease hysteresis in comparison with thin films. Overall, our findings pave the way for a fast transfer of combinatorial thin film research towards bulk-like materials for magnetic Heusler alloys.

## Methods

A free standing thin film of Ni_39.4_Co_9.2_Mn_32.3_Al_19.1_ with a thickness of 200 nm was prepared by magnetron co-sputtering (see Teichert *et al*. for experimental details^8^). For the melt-spinning an ingot of the nominal composition Ni_40_Co_10_Mn_30_Al_20_ has been produced by arc melting. Melt-spun ribbons were prepared by rapid quenching of the melted (T = 1910 K) ingots using Ar overpressure of p = 300 mbar on a copper wheel with a gap between wheel surface and nozzle of d = 600 µm. The linear velocity of the rotating wheel was v = 16 ms^−1^. The quenched ribbons have a thickness between 50 and 100 µm, a width of approximately 4 mm and a length of several centimetres. Heat treatment of the ribbons has been done under argon atmosphere at T = 850 °C for a duration of t = 0.25, 1, 2, 8, 16, 24 and 50 h. After the annealing time, the sample were cooled under Ar flow with an approximate cooling rate $\dot{T}$ > 100 Kmin^−1^.

The composition of the as-cast sample was checked with inductively coupled plasma-optical emission spectrometry (ICP-OES), the one of the ribbon samples with energy dispersive X-ray spectroscopy (EDX) in a scanning electron microscope (Zeiss Leo Gemini 1530). The determined composition was Ni_40.9_Co_10.8_Mn_29.3_Al_19.1_. The structure was investigated with X-ray diffraction using a Philips X´Pert Plus Diffractometer (Co-K_α_, λ = 1.789 Å) in Bragg-Brentano-geometry at room temperature. To determine ordering from the diffraction data, the height ratio between ordering and fundamental peak, related to the theoretical peak intensity ratio based on atomic form factors, unit cell, multiplicities, Debye-Waller and Lorentz-factor, needs to be considered. In the case of B2 ordering, which is the primary ordering effect in the present alloy, we use the expression:

1 $${S}^{2}(B2)=\,{\frac{I(200)}{I(400)}}_{measured}{/}{\frac{I(200)}{I(400)}}_{theoretical}$$

The subordering in B2 explored in the numerical simulations is not accessible by X-ray diffraction and therefore subject to ab initio calculations.

Both magnetisation and the heat capacity measurements were carried out using a Quantum Design PPMS with a vibrating sample magnetometer (VSM) and a heat capacity inset, respectively. The heat capacity was determined using the 2τ method^38^. The entropy change ∆S_T_ was calculated from isothermal magnetisation curves under magnetic field change of ∆µ_0_H = 0–2 T, in steps of µ_0_H = 0.1 T, using the thermodynamic Maxwell relation^39^:

2 $${\rm{\Delta }}{S}_{T}={\mu }_{0}{\int }_{{H}_{0}}^{{H}_{1}}{(\frac{\partial M}{\partial T})}_{H}\,dH$$

The adiabatic temperature change ∆T_ad_ was calculated using the heat capacity and entropy change data^40^:

3 $${\rm{\Delta }}{T}_{ad}=-\,\frac{T}{{c}_{p,H=0T}}{\rm{\Delta }}{S}_{T}$$

All quantum-mechanical simulations were performed with the Vienna *ab initio* simulation package (VASP)^41–43^ employing projector augmented wave (PAW) potentials^44^. The generalized gradient approximation (GGA) as parameterized by Perdew, Burke and Ernzerhof (PBE)^45^ was used for the exchange-correlation functional. An energy cut-off of 450 eV was chosen for the plane-wave basis. For the sampling of the Brillouin zone, the Monkhorst-Pack scheme^46^ with a k-point grid of 20 × 20 × 20 was used. An energy tolerance of 10^−7^ eV was used as a convergence criterion for the self-consistent electronic loop. All atomic positions were relaxed until the residual forces acting on the atoms are less than 10^−3^ eV/Å. The calculations were performed for ordered spin alignments relevant at application temperatures, while a consideration of all the paramagnetic degrees of freedom expected under annealing conditions would be beyond the scope of this paper.

### Data availability

The datasets generated and analysed during the current study are available from the corresponding author on reasonable request.

## Electronic supplementary material

Supplementary information
