# Supplementary material for: Role of disorder when upscaling magnetocaloric Ni-Co-Mn-Al Heusler alloys from thin films to ribbons
Source: Sci Rep. 2018 Jun 14;8:9147. doi: 10.1038/s41598-018-27428-8 (PMC6002362; doi:10.1038/s41598-018-27428-8)
Supplement: Supplementary file 1 — Supplementary information [file 41598_2018_27428_MOESM1_ESM.pdf]

Supplementary information
Role of disorder when upscaling magnetocaloric Ni-Co-Mn-Al Heusler alloys from thin films to ribbons

B. Weise, B. Dutta, N. Teichert, A. Hütten, T. Hickel and A. Waske

In supplementary Figure S1 the annealing time dependence of the transition temperatures for both martensite- and austenite transition (T_M, T_A), are shown. Additionally the start- and finish temperatures for martensite- and austenite transition (M_s, M_f, A_s and A_f) are given. It can be seen clearly that already a short annealing (t = 0.25 min) leads to a drastic increase in all transition temperatures, while the changes are more subtle for longer annealing times.

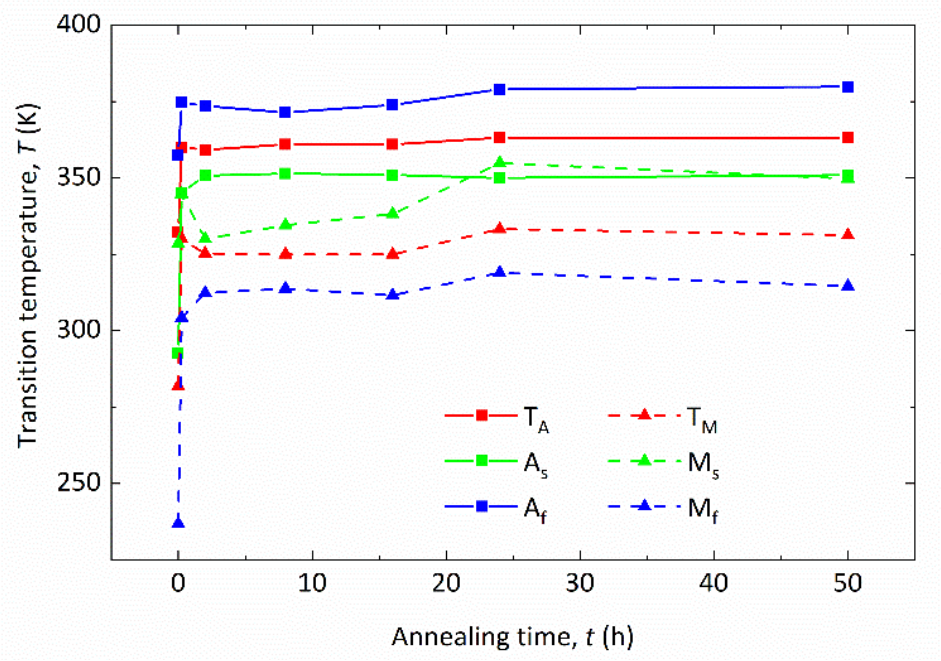

Figure S1: Dependence of the transition-, start- and finish temperatures of the austenite and martensite transition of the melt-spun ribbons on the annealing time.
